# A Case of Delayed Diagnosis of Bilateral Ureteral and Bladder Injury after Laparoscopic Hysterectomy: An Unusual Complication

**DOI:** 10.1155/2012/817010

**Published:** 2012-11-06

**Authors:** Maximilien C. Goris-Gbenou, Nicolas Arfi, Abdel Mitach, Sheer Rashed, Jean-Gabriel Lopez

**Affiliations:** Department of Urology, Centre Hospitalier de Valence, 179 boulevard Maréchal Juin, 26000 Valence, France

## Abstract

The incidence of ureteral and bladder lesions after laparoscopic hysterectomy is the most encountered urinary complication in gynaecological surgery. We report the unusual case of 42-year-old woman who had a delayed diagnosis of bilateral ureteral injury associated with bladder lesion and loose of vaginal suture after undergoing laparoscopic hysterectomy for uterine adenomyosis.

## 1. Introduction

The incidence of ureteral and bladder lesions after hysterectomy is 0.3% to 4.3% [1, 2]. Such lesions usually provoke acute renal insufficiency requiring nephrostomy [1]. They are easier to manage when detected intraoperatively than later [3, 4]. Late diagnosis of ureteral lesions, with or without bladder injury, requires further intervention and is the reason for many medical claims [5, 6].

## 2. Case Presentation

A 42-year-old woman, with no medical history, was admitted to department of gynaecology after suffering from chronic pelvic pain, metrorrhagia, and dyspareunia for several months. Uterine adenomyosis was diagnosed on magnetic resonance imaging (MRI) of the pelvis. A laparoscopic hysterectomy was performed with no intraoperative complications or surgery-related technical problems. At the close of the procedure, the urinary catheter drained clear urine. It was removed on postoperative day 2. The patient recovered spontaneous micturition but complained of urinary incontinence on day 3. She was discharged on day 4 (creatinine, 82 micromoles/L; no inflammatory syndrome). 

**Figure 1:**
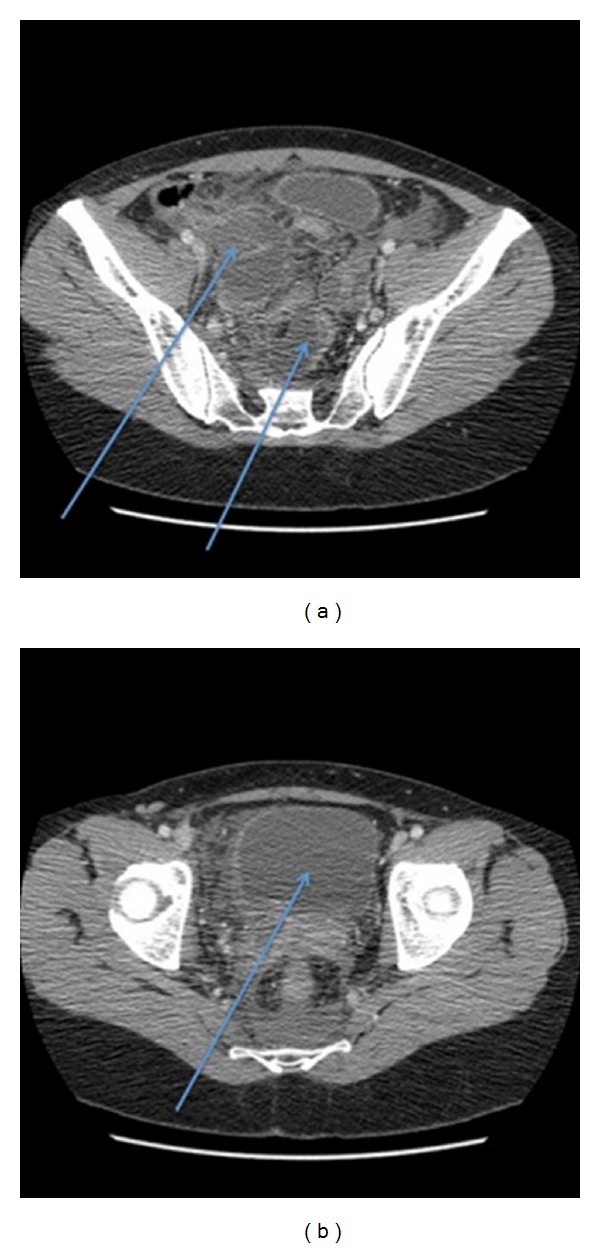
CT scan of the abdomen and pelvis, with contrast agent injection, on postoperative day 8, no excretory phase images. No urologic lesions were detected. (a) Presence of 2 pelvic collections (45 and 35 mm) and (b) full bladder.

On postoperative day 8, the patient was readmitted with abdominal pain, inability to pass stools and gas, and vomiting. She complained of vaginal loss and persistent urinary incontinence but had no fever. The clinical examination was suggestive of bowel obstruction, with muscle contraction on abdominal palpation. The surgical scars were healthy. Laboratory findings were leucocytosis, 11.5 g/L; C reactive protein, 12 mg/L; and creatinine, 65 micromoles/L. A computed tomography (CT) scan of the abdomen and pelvis, with injection of contrast agent, revealed bilateral hypotonia of the pyelocaliceal cavities, dilatation of the right lumbar ureter, a full bladder, peritoneal effusion (fluid collection of 45 and 35 mm), and small bowel dilatation with no mechanical obstruction (Figure 1). The obstructive syndrome persisted. 

**Figure 2:**
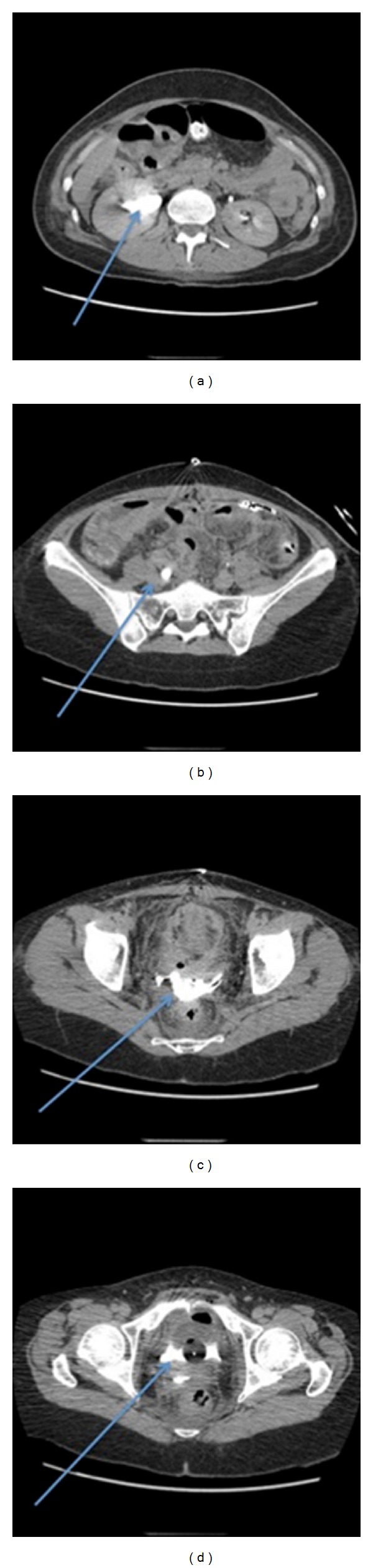
CT scan of the abdomen and pelvis on postoperative day 17. (a) Excretory phase. The arrow shows dilatation of the right pyelocaliceal cavities. (b) Dilatation of the right ureter. (c) and (d) Urinoma with extravasation of contrast agent into the pelvis.

Initial management was conservative on the gastrointestinal surgeon's advice. A gastric catheter was placed in the fasting patient; 1.5 L were collected in 24 hours. The patient had dysuria. Kidney function was impaired (increased creatinine, 268 micromoles/L with decreased creatinine clearance at 32 mL/min). Fever and persistent contraction on abdominal palpation prompted an exploratory examination on day 17, which revealed peritonitis; diffuse adhesions of the ileum, and intraperitoneal effusion of a fluid resembling urine. A 1.5 × 4 cm inflammatory lesion of the Douglas pouch was excised and, on pathological examination, proved to be due to endometriosis and an abscess. The vaginal vault was open and had required closure by 2 stitches. The abdominal cavity was drained with a Shirley sump drain. The urinary catheter drained no urine during the first hours following the intervention, but increasing the intravenous infusion and injection of diuretic led to the appearance of urine in the Shirley drain. The urologist on duty requested a methylene blue test, which proved positive for the vagina and Shirley drain. An abdomen and pelvis CT scan confirmed bilateral ureteral injury (Figure 2).

**Figure 3:**
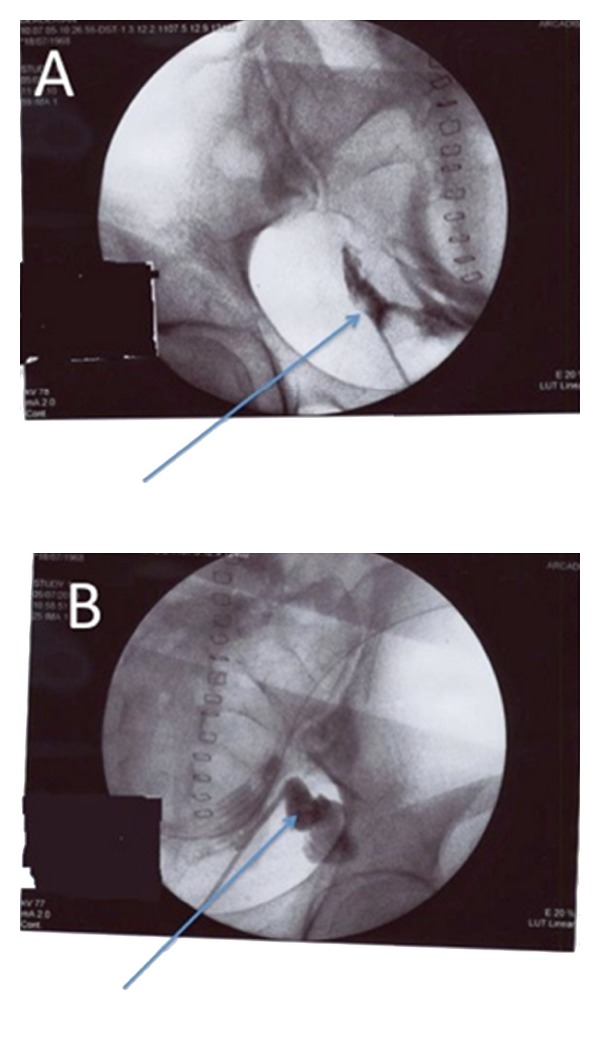
Retrograde ureteropyelography on postoperative day 17 showing extravasation of contrast agent from both pelvic ureters ((A) right, (B) left).

In the absence of pyelocaliceal cavity dilatation, we did not perform percutaneous bilateral nephrostomy but attempted insertion of ureteral endoprostheses after bilateral ureteropyelography. Cystoscopy revealed that the posterior bladder wall was perforated 3 cm above the intertrigonal ridge. Retrograde ureteropyelography confirmed bilateral ureteral injury (Figure 3). We were unable to insert hydrophilic wire guides into both ureters to allow urine drainage. 

The patient's clinical condition warranted a second laparotomy. Surgical exploration revealed partial ligature and iatrogenic transection of the ureters at the level of the uterine arteries. The ureters leaked. The discovery of adnexal ischemia and ovarian necrosis prompted a left ovariectomy. A Politano-Leadbetter bilateral ureter reimplantation was performed after suture of the bladder breach. Hyperdiuresis (6 L/24 hours) on postoperative day 1 was followed by a favourable postoperative clinical course. Normal intestinal transit was recovered on day 5. Creatinine level was 70 micromoles/L. Cystography showed minimal posterior leakage on day 15 and none on day 25. The ureteral endoprostheses were removed during postoperative month 2. Pathology revealed a normal urothelium with acute inflammation and necrosis at the ureteral margins, and inflammation and acute ischemia of the left ovary. 

Two years after surgery, the patient was symptom-free. The abdomen and pelvis CT scan and urologic parameters were normal. Creatinine was 68 micromoles/L.

## 3. Discussion

To our knowledge, late diagnosis of bilateral iatrogenic ureteral injury and injury to the bladder during laparoscopic hysterectomy has not been reported. Diagnosis was delayed in our patient because of leakage leading to partial conservation of micturition. Urine loss via the vagina was due to a loose vaginal suture. A urinoma had formed because the bladder breach and vaginal opening did not allow complete continuous urine evacuation; the urethrocutaneous fistula was formed after vaginal wound closure and catheter placement.

Such dual injury should be routinely sought when faced with an acute abdominal syndrome after laparoscopic hysterectomy. The diagnosis should be established by the methylene blue test, a CT scan, and/or retrograde urography, and the placement of a double-J catheter should be attempted. In the event of pyelocaliceal cavity dilatation, nephrostomy will be preferred. Bilateral ureteral reimplantation with bladder suture is feasible and represents standard first-line management after failure of a minimally invasive procedure or after renal drainage. Omental wrapping can also be used to effectively treat ureteral injury [7, 8]. During exploratory laparotomy on the 17th postoperative day, we focused our attention on the ureteral and/or bladder injury because of the frequency of these lesions in the literature [9]. 

A recent published study by Janssen in 37 patients during a 20-year period [10] have shown that main predisposing factors, retrospectively assessed, were: incomplete learning curve, insufficient applied technique such as coagulation of the uterine artery without the use of a uterine manipulator, and/or from the contralateral side and/or without previously performed ureterolysis in case of distorted anatomy. In this study, only one ureter injury was diagnosed during the laparoscopy hysterectomy; the mean time to diagnose the injury was 29 days.

The urinary tract injury should be investigated in case of acute abdominal syndrome after laparoscopic hysterectomy even occurring late.
